# Imaging of post-infarction myocardial inflammation with hybrid FDG PET/MR: feasibilty and preliminary findings in a canine model

**DOI:** 10.1186/1532-429X-17-S1-Q19

**Published:** 2015-02-03

**Authors:** Gerald Wisenberg, Yoko Mikami, James A White, Kimberley Blackwood, Edward J Tweedie, Terry R Thompson, Frank S Prato

**Affiliations:** 1 Medicine, London Health Sciences Centre, London, ON, Canada; 2 Medicine, University of Calgary, Calgary, AB, Canada; 3 Medical Biophysics, Lawson Health Research Institute, London, ON, Canada

## Background

An understanding of inflammation following myocardial infarction may be of importance in the development of novel therapeutics to limit the development of heart failure following myocardial injury. However, the quantification of inflammation in this setting continues to be challenging. Hybrid FDG PET/MR may offer a non-invasive in vivo solution through intrinsic registration of these complementary modalities. This study sought to validate its use in a canine model of myocardial infarction (MI).

## Methods

Twenty dogs underwent a left thorocotomy with permanent or transient 3 hour ligation of the left anterior descending to induce MI. Hybrid PET/MR was performed on day 7 in all animals and out to day 21 in a subset of animals following suppression of basal myocardial glucose metabolism with a lipid infusion. Blinded analysis of fused imaging quantified FDG activity within and beyond MR-defined infarct zones. A detailed serial imaging evaluation and histologic comparison was also performed. Nine of these animals, sacrificed at various time points from day 7 through 21, also were the subject of ex vivo tissue analysis to establish correlations between concentrations of FDG, Indium-111, which had been used to label white blood cells injected 24 hours prior to sacrifice, and Tc99^mTc^-DTPA, used to mirror the distribution of Gd-DTPA.

## Results

All animals survived and completed imaging. Infarction was achieved in 18/20 subjects. Fused PET/MR yielded an imaging correlation of elevated FDG tracer activity within regions of myocardial infarction but reduced activity in regions of microvascular obstruction. A modest elevation was also seen in remote non-infarcted tissue. Animals studied out to day 21 showed signficant associations between tracer activity and adverse remodeling based on MR-derived left ventricular volumes. Histopathology correlated tracer activity to inflammatory celular infiltrate. In the 9 animals subject to ex vivo tissue analysis, there was a good correlation between FDG vs. Indium concentrations across the range of Tc-DTPA values, except in regions of microvascular obstruction and low Tc-DTPA, where Indium levels were much higher than those of FDG.

## Conclusions

Hybrid PET/MR visualizes inflammatory activity early following MI and provides intrinsic registration to MR tissue imaging, which defines regions of infarct and microvascular obstruction. Reduced FDG activity within regions of microvascular obstruction may be secondary to limited delivery of tracer to these regions following bolus injection of the tracer. Our preliminary findings suggest a potential for evaluating infarct related inflammation and to provide associations to downstream adverse remodeling. Future studies using this novel imaging platform are justifed to explore such relationships and to determine if a constant infusion of both MR and FDG tracers may allow more precise quantification of the degree of inflammation within regions of microvascular obstruction.

## Funding

1) Ontario Research Fund: Imaging in Cardiovascular Therapeutics Grant

2) Canadian Institutes of Health Research grant; IMAGE-HF

3) Program of Experimental Medicine (POEM), Department of Medicine, University of Western Ontario

4) Canadian Foundation for Innovation and the Ministry of Research and Innovation of the Province of Ontario: Infrastructure grant.

**Figure 1:**
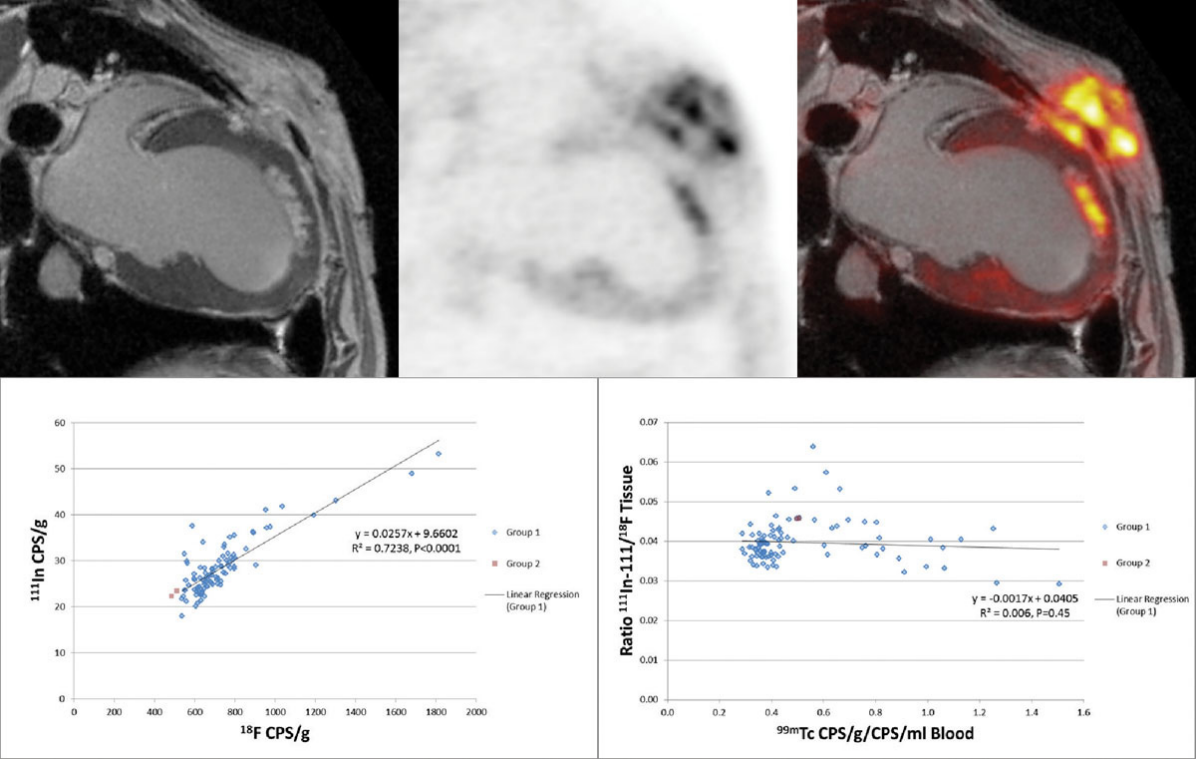
Post mortem PET/MRI images obtained 14 days after a three hour LAD occlusion followed by reperfusion. The late enhanced MR image (left) demonstrates an antero-apical subendocardial infarct. The non-transmural endocardial infarction showed no evidence of microvascular obstruction assessed from the in vivo LGE 3-D data set, consistent with visual inspection of the post mortem LGE images. Enhanced FDG activity is seen within the infarct region (centre-PET image alone right-fused PET/MR). The regression of ^111^In with ^18^F was strong (R^2^ = 0.724) and the slope significantly positive (0.0257 ± 0.0017, p<0.0001).

**Figure 2:**
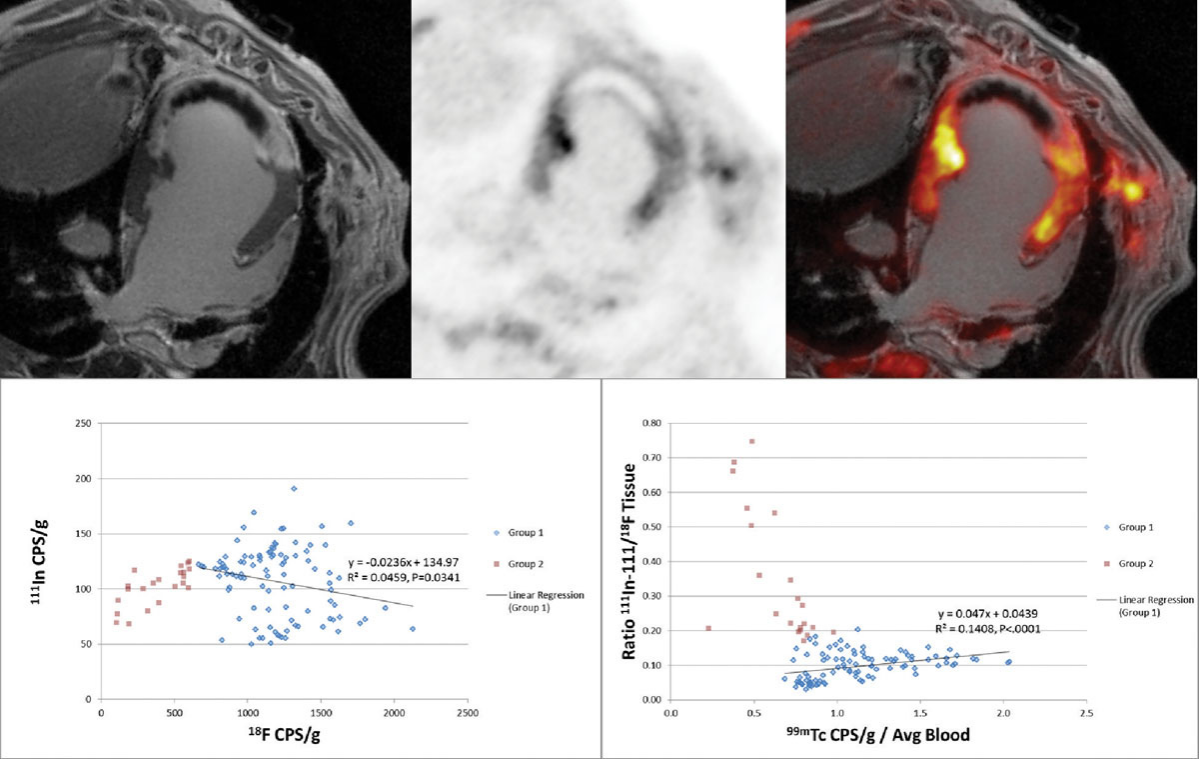
Post-mortem PET/MRI images from subject with permanent LAD occlusion imaged 7 days post MI. The images demonsrate both enhanced Gd-DTPA and FDG uptake surrounding a large central zone of microvascular obstruction (MO), suggesting minimal inflammatory cell activity in that region. However, analysis of the ex vivo tissue demonstrates a dissociation between Indium and FDG concentrations in tissue obtained from the MO zone. In some samples, Indium levels were up to 7 times higher, suggesting penetration of the labeled white blood cells injected 24 hours earlier, but minimal FDG following a single bolus injection. Therefore, FDG imaging may signficantly underestimate the degree and extent of inflammatory cell activity in the setting of MO.

